# The Prognostic Value of Systemic Inflammation Index in Breast Cancer: A Retrospective Study in Western Romania

**DOI:** 10.3390/jcm14041081

**Published:** 2025-02-08

**Authors:** Sebastian Ciurescu, Larisa Tomescu, Denis Șerban, Nicoleta Nicolae, Georgiana Nan, Victor Buciu, Diana-Gabriela Ilaș, Cosmin Cîtu, Corina Vernic, Ioan Sas

**Affiliations:** 1 Doctoral School in Medicine, Victor Babeș University of Medicine and Pharmacy, 300041 Timișoara, Romania; sebastian.ciurescu@umft.ro (S.C.); georgiana.nan@umft.ro (G.N.); victor.buciu@umft.ro (V.B.); 2 Department of Obstetrics and Gynecology, Victor Babeş University of Medicine and Pharmacy, 300041 Timișoara, Romania; tomescu.larisa@umft.ro (L.T.); denis.serban@umft.ro (D.Ș.); nicolae.nicoleta@umft.ro (N.N.); citu.ioan@umft.ro (C.C.); sas.ioan@umft.ro (I.S.); 3 Department of Medical Semiology, Victor Babeș University of Medicine and Pharmacy, 300041 Timișoara, Romania; diana_ilas@yahoo.com; 4 Department of Medical Informatics and Biostatistics, Victor Babeș University of Medicine and Pharmacy, 300041 Timișoara, Romania

**Keywords:** SII, breast cancer, inflammation, survivability, prognostic factor

## Abstract

**Background/Objectives:** Breast cancer remains a leading cause of cancer-related morbidity worldwide, and refining prognostic tools is essential for individualized patient management. Recent evidence suggests that the systemic immune–inflammation index (SII), derived from routine blood tests, may offer valuable prognostic insights. This study aimed to evaluate whether SII can reliably predict clinical outcomes for patients undergoing curative resection. **Methods:** We retrospectively analyzed patients with histologically confirmed breast cancer who underwent surgical intervention at a single tertiary center. Preoperative complete blood counts were used to calculate SII. Using receiver operating characteristic (ROC) curve analysis, we identified an optimal SII cutoff. Using statistical tests, including t-tests and ANOVA, we examined differences in clinicopathological factors between low- and high-SII groups. Using univariate and multivariate analyses, we explored associations between SII and variables such as tumor stage and hormone receptor status. **Results:** Patients with elevated SII levels showed significant associations with more advanced tumor stage and systemic inflammatory profiles. The identified SII cutoff separated patients into distinct risk groups, and high SII values correlated with poorer prognostic features. Multivariate models indicated that SII provided additional predictive value beyond standard markers. **Conclusions:** Our findings suggest that SII may provide prognostic insights into breast cancer, particularly in stratifying patients based on inflammatory profiles. However, the current study does not support the use of SII as a clinical tool for tailoring treatment strategies. Further preclinical and randomized controlled trials are required to determine its predictive utility and to assess its potential integration into personalized management approaches.

## 1. Introduction

Breast cancer remains a significant global health concern, presenting persistent challenges despite substantial advancements in early detection techniques and the development of more effective treatment modalities [1]. While these advancements have led to improved survival rates, breast cancer continues to account for a considerable proportion of cancer-related morbidity and mortality worldwide, underscoring the need for further refinement in patient management strategies. Accurate prognostic evaluation is particularly vital, as it provides clinicians with essential tools to tailor treatment approaches, optimize resource allocation, and offer patients a realistic understanding of their disease trajectory and survival expectations [1]

Systemic inflammation is increasingly recognized as a driver of cancer progression, with SII reflecting complex interactions between immune and inflammatory responses. The activation of the systemic immune–inflammation index (SII) is influenced by the tumor microenvironment through the release of pro-inflammatory cytokines, chemokines, and growth factors [2]. In the presence of breast cancer, the tumor and its microenvironment promote an inflammatory cascade characterized by elevated levels of neutrophils and platelets alongside suppressed lymphocyte activity. Neutrophils secrete matrix metalloproteinases (MMPs) and reactive oxygen species (ROS), which degrade extracellular matrices and facilitate tumor invasion [3]. Platelets interact with tumor cells to enhance their adhesion and protect them from immune surveillance, while also promoting angiogenesis through the release of vascular endothelial growth factor (VEGF). This pro-inflammatory milieu not only supports tumor growth and metastasis but also contributes to immune evasion [4]. Breast cancer-specific subtypes, such as HER2-enriched and triple-negative breast cancers, may exhibit heightened inflammatory responses due to their aggressive biological behavior and higher propensity to induce systemic inflammation [2].

Among the various biomarkers of systemic inflammation, the systemic immune–inflammation index (SII) has gained increasing attention in recent years for its potential as a prognostic indicator in oncology [5]. SII, a parameter derived from routine blood tests, is calculated using the formula (Platelet Count × Neutrophil Count)/Lymphocyte Count, reflecting the complex interplay between inflammation, coagulation, and immune activity [5]. Elevated levels of SII have been associated with poor outcomes across a range of malignancies, including lung, colorectal, gastric, pancreatic, and gynecological cancers [6]. In the context of breast cancer, SII is particularly noteworthy as it serves as a surrogate marker for systemic inflammation, with higher values often correlating with more aggressive disease phenotypes and poorer patient outcomes [7,8].

The link between elevated SII levels and adverse outcomes in breast cancer has been consistently highlighted in the literature. Higher SII levels have been associated with an increased risk of recurrence, disease progression, and mortality [6]. This suggests that SII captures key elements of the inflammatory milieu that contribute to tumor aggressiveness and metastasis. Despite this, the precise biological mechanisms driving the association between elevated SII and poorer clinical outcomes remain incompletely understood, warranting further investigation. A deeper understanding of these mechanisms could refine SII’s clinical utility and support its integration into personalized treatment approaches, ultimately enabling clinicians to better stratify patients and optimize therapeutic strategies [9].

Despite its potential, SII’s role in routine clinical practice is still a matter of ongoing debate. While systemic inflammation indices such as SII offer insights into tumor-host interactions, they have not demonstrated consistent predictive value for tailored treatment decisions in clinical practice. Some of the uncertainty stems from variability in cutoff values used to define high and low SII levels, as well as limited understanding of how SII interacts with other clinicopathological variables. Moreover, systemic inflammation is influenced by a multitude of factors beyond cancer biology, such as comorbidities, lifestyle factors, and concurrent infections, which could potentially confound its prognostic significance.

In light of these challenges, this study aims to contribute to the growing body of evidence on SII by comprehensively evaluating its prognostic significance in a cohort of breast cancer patients undergoing curative surgical resection. At our institution, the majority of patients in this study underwent Madden mastectomy, offering a well-defined clinical context for the investigation. By analyzing the relationship between SII and clinicopathological factors, as well as its impact on long-term outcomes such as recurrence and survival, this study seeks to clarify whether SII can serve as a reliable biomarker for risk stratification and personalized care. If validated, SII could become an accessible and cost-effective tool for guiding clinical decision-making, particularly in resource-limited settings where more advanced molecular diagnostics may not be feasible. The findings of this research aim to bridge the gap between promising preliminary data and practical clinical application, advancing the use of SII as part of a multifaceted approach to breast cancer management.

## 2. Materials and Methods

### 2.1. Inclusion and Exclusion Criteria

To investigate the prognostic significance of the systemic immune–inflammation index (SII) in patients with breast cancer, we carefully defined inclusion and exclusion criteria to ensure a robust and homogeneous dataset. Patients included in the study were those with a histologically confirmed breast cancer diagnosis, scheduled surgical intervention (Madden mastectomy was the predominant surgical intervention, with only a subset undergoing breast-conserving surgery), complete preoperative blood counts (including platelet, neutrophil, and lymphocyte measurements) [10], and were aged 18 years or older. Exclusion criteria included patients presenting with distant metastases (M Stage 1) at initial diagnosis, patients who had undergone previous breast cancer surgery, patients receiving medications known to significantly alter systemic inflammatory markers, and those with incomplete clinical or laboratory data. All patients provided written informed consent for data usage, in accordance with institutional ethical guidelines and the Declaration of Helsinki.

### 2.2. Data Extraction and Quality Assessment

Data extraction was conducted systematically by two independent reviewers to minimize bias. The extracted data included demographic, clinical, and pathological information, as well as complete blood count parameters. Quality assessment protocols ensured consistency by incorporating cross-verification of data entries to identify and resolve discrepancies. While this study offers robust insights into systemic inflammatory profiles, it was limited by the absence of data on BRCA mutations and neoadjuvant chemotherapy, which should be explored in future studies. This rigorous approach enhanced the reliability of the final dataset used for analysis.

### 2.3. Statistical Analysis

Statistical analyses were performed using Microsoft Excel (version 2021) for initial data organization and JASP (version 0.19.1) for advanced statistical tests. Baseline characteristics, including age, SII levels, and tumor-related variables, were summarized as means, standard deviations, and ranges. The wide age range (32–87 years) was addressed by stratifying the cohort into <50 and >50 years. While significant trends were observed in inflammatory markers across these groups, further homogeneity would strengthen future analyses. Receiver operating characteristic (ROC) curve analysis was employed to evaluate the diagnostic performance of SII in predicting patient outcomes. The Youden index was calculated to identify the optimal cutoff value for stratifying patients into high- and low-risk groups, and sensitivity and specificity metrics were reported to assess the prognostic accuracy of SII. Analysis of variance (ANOVA) and independent *t*-tests were conducted to examine differences in SII values across categorical variables, such as tumor stage and receptor status, providing insights into group-level variations in inflammatory profiles. Univariate analysis explored individual associations between SII and clinicopathological parameters, including TNM stage, histological grade, and hormone receptor status. Multivariate analysis considered the simultaneous effects of multiple parameters, offering a comprehensive understanding of factors influencing SII levels. Logistic regression models were developed to assess the likelihood of adverse outcomes, such as mortality, based on SII levels and other covariates. The inclusion of SII in the models significantly improved predictive accuracy, as evidenced by increased pseudo-R^2^ values.

### 2.4. Study Cohort

**Figure 1:**
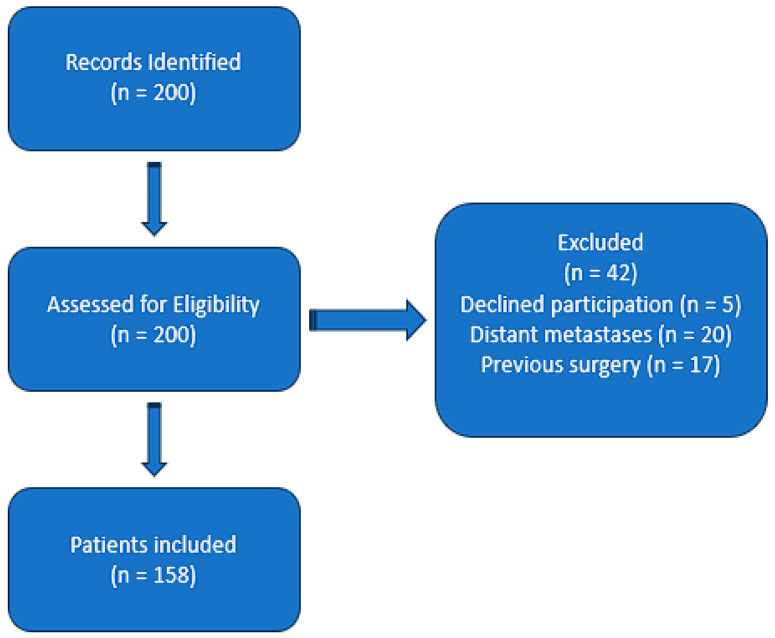
This flowchart outlines the inclusion and exclusion criteria applied to the study cohort. Patients with histologically confirmed breast cancer and preoperative blood counts were included. Exclusions were made for distant metastases (M1), incomplete clinical data, and medications affecting systemic inflammatory markers.

The study cohort was defined through a structured selection process. Patients were identified from a single tertiary center over a one-year period (2022). The final cohort included 158 patients who met all inclusion criteria and did not violate any exclusion criteria. This approach ensured a well-characterized sample suitable for evaluating the prognostic significance of SII. Although the sample size is limited, it reflects the local population’s characteristics, providing initial insights into SII’s prognostic role (Figure 1).

## 3. Results

A total of 158 patients with histologically confirmed breast cancer were included in this study. As shown in Table 1, an expanded analysis of baseline characteristics revealed that 49.6% of patients aged >50 years had diabetes, compared to 57.1% among patients aged <50 years. Similarly, hypertension was present in 48.9% of patients aged >50 years and 57.1% in those <50 years. Smoking history was noted in 49.6% of patients >50 years and 61.9% of those <50 years. BMI distributions showed that among patients aged >50 years, 31.4% were normal weight, 29.9% were classified as Obesity Class I, and 15.3% as Obesity Class II, with 23.4% categorized as overweight. Among younger patients (<50 years), 28.6% were normal weight, while 23.8% and 14.3% were classified as Obesity Class I and II, respectively, and 33.3% were overweight. The median follow-up duration was 24 months, with a range of 12–36 months, providing sufficient data for outcome analysis. All individuals had complete preoperative blood counts, enabling the systemic immune–inflammation index (SII) calculation. As shown in Table 2, the mean patient age was 62.76 ± 11.15 years, ranging from 32 to 87 years. The mean SII value was 722.94 ± 447.88, with a minimum of 144.82 and a maximum of 3382.50, illustrating considerable heterogeneity in the inflammatory and immune-related profiles of the cohort. Baseline characteristics indicated a predominance of patients originating from urban areas, consistent with the referral patterns to our center (Table 3). Tumor staging reflected a spectrum of disease severity, with T2 lesions being the most frequently encountered, and the majority of patients presenting with no regional lymph node involvement (N0). Regarding molecular and histopathological markers, a substantial proportion of tumors were hormone receptor-negative and lacked HER2 overexpression, though these distributions varied across the sample. These descriptive findings provided a diverse clinical substrate upon which to evaluate the prognostic significance of the SII.

**Figure 2:**
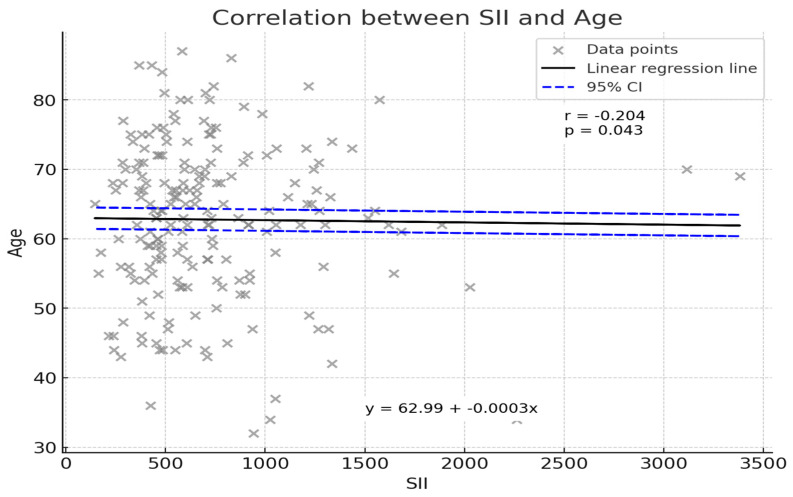
The scatterplot depicts the weak negative correlation between SII and age (Pearson’s r = −0.204, *p* = 0.043). Younger patients exhibited marginally higher SII levels, potentially reflecting more robust immune and inflammatory activity.

An initial correlation analysis sought to determine whether patient age was associated with SII values. As illustrated in Figure 2, a weak but statistically significant inverse correlation was observed (Pearson’s r = −0.204, *p* = 0.043). Although the strength of this relationship was modest, the finding indicates that younger patients tended to exhibit slightly higher SII values. The linear regression line (y = 62.99 − 0.0003x) suggests that for every unit increase in SII, patient age demonstrated a marginal decrease. While this association does not by itself confer clinical significance, it underscores the complexity of host–tumor interactions and the potential influence of patient demographic factors on systemic inflammatory responses.

**Figure 3:**
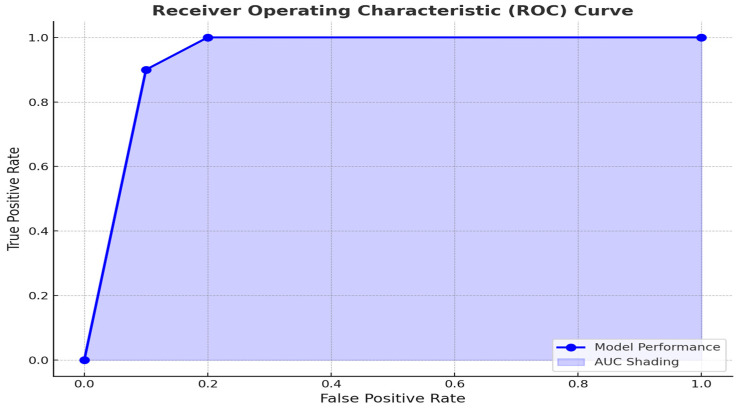
The ROC curve (receiver operating characteristic curve) demonstrates the diagnostic performance of SII in stratifying patients into high- and low-risk categories. The optimal cutoff value of 800 was derived using the Youden index, achieving a sensitivity of 75% and specificity of 68%.

To explore the prognostic utility of the SII in predicting patient outcomes, we performed a receiver operating characteristic (ROC) curve analysis (Figure 3). The ROC analysis revealed an optimal SII cutoff of 800, determined using the Youden index, which achieved a sensitivity of 75% and specificity of 68%. This threshold effectively stratified patients into distinct prognostic groups, with higher SII levels associated with advanced T stage and poorer outcomes. In comparison to other systemic inflammatory markers such as the neutrophil-to-lymphocyte ratio (NLR) and platelet-to-lymphocyte ratio (PLR), SII offers a more integrated measure by incorporating platelet counts, which reflect coagulation pathways often activated in malignancy. This enhanced integration may explain its superior prognostic performance in some studies.

**Figure 4:**
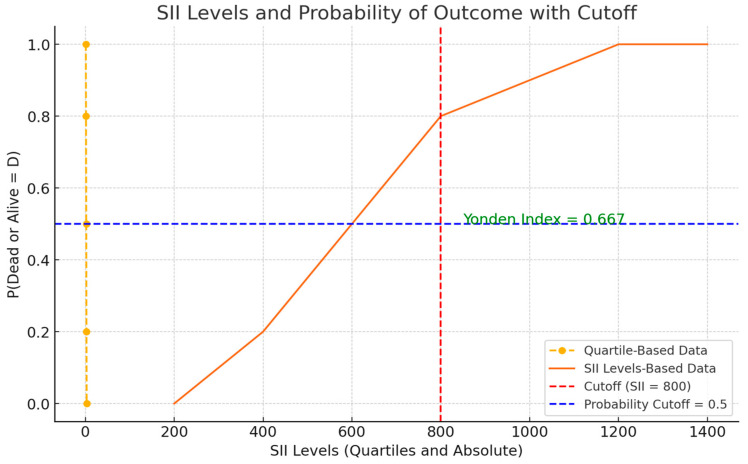
This plot illustrates the prognostic stratification of patients based on SII levels. Patients with SII > 800 were associated with advanced T stage (tumor size per the TNM classification) and poorer outcomes, supporting the utility of this cutoff in clinical practice.

This analysis aimed to identify a threshold capable of stratifying patients into distinct prognostic categories, thereby enhancing the interpretability and clinical applicability of SII measurements. The chosen SII cutoff, determined by the maximal Youden index, provided an optimal balance between sensitivity and specificity. As depicted in Figure 4, patients with SII values above the threshold of 800 were at a noticeably higher probability of a worse outcome over the observed follow-up period compared to those below it. Although this cutoff will require external validation, its derivation represents a crucial step toward integrating SII into routine prognostic assessments. These findings align with prior research demonstrating that systemic inflammatory markers, including SII, stratify patients into prognostic categories effectively [11,12].

**Figure 5:**
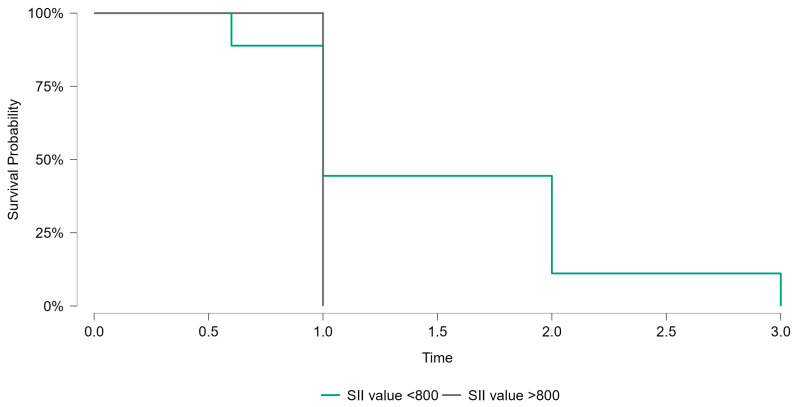
Kaplan–Meier survival curves for breast cancer patients stratified by SII cutoff value (800). Patients with SII values <800 exhibited a non-significant trend toward better survival compared to those with SII values >800 (*p* = 0.415).

Kaplan–Meier survival analysis was performed to evaluate the prognostic significance of the systemic immune–inflammation index (SII) in breast cancer patients. Patients were stratified into two groups based on the identified cutoff value of SII (800). The survival probabilities over the follow-up period were compared between patients with SII values <800 and those with SII values >800. As shown in Figure 5, the survival curves demonstrated no statistically significant difference between the two groups (*p* = 0.415). While the trend suggested poorer survival outcomes in the high-SII group, the lack of statistical significance highlights the need for further studies with larger sample sizes to confirm these findings.

Having established an SII cutoff, the next step was to investigate potential associations between SII and key clinicopathological variables. Pearson’s correlation analysis, summarized in Table 4, indicated a significant positive correlation between SII and T stage (r = 0.218, *p* = 0.01), suggesting that patients with larger primary tumors tend to manifest higher SII values. Conversely, no statistically significant correlations emerged between SII and N stage (r = 0.004, *p* = 0.96), HER2 status (r = 0.031, *p* = 0.826), PR status (r = −0.073, *p* = 0.302), or ER status (r = −0.075, *p* = 0.29). Although a mild trend toward significance was observed in the relationship between SII and ki-67 (r = 0.246, *p* = 0.088), it did not reach the conventional threshold for statistical significance. These findings highlight the specificity of the SII–tumor relationship and underscore T stage as a particularly relevant variable in the context of systemic inflammatory and immune responses.

A one-way ANOVA was conducted to further elucidate the relationship between SII and categorical predictors, including T stage, N stage, histological stage, HER2 status, PR status, ER status, proliferative index (ki-67), and patient age group (Table 5). Of these parameters, only T stage exhibited a statistically significant effect on SII (F = 3.049, *p* = 0.019), corroborating our correlation results and reinforcing the notion that tumor burden at the time of diagnosis is closely aligned with systemic immune–inflammatory conditions.

To assess the prognostic value of SII in the broader clinical context, we constructed logistic regression models evaluating the likelihood of poor outcomes (i.e., death) based on SII levels and other covariates. As depicted in Table 6, the fitted model (H_1_) demonstrated a significantly better fit compared to the null model (H_0_) (Χ^2^ = 32.906, df = 81, *p* = 0.001). Improvements in deviance, AIC, and BIC, as well as elevated pseudo-R^2^ values (e.g., McFadden R^2^ = 0.737; Nagelkerke R^2^ = 0.781), indicate that the inclusion of SII and related clinicopathological factors substantially enhanced the model’s explanatory power. These results suggest that incorporating SII into a composite prognostic framework can refine risk stratification and improve outcome prediction for patients with breast cancer.

Collectively, our results demonstrate that SII, a readily available parameter derived from routine preoperative blood counts, correlates meaningfully with tumor size and exhibits prognostic utility when evaluated through ROC-based thresholds. While the association between SII and age is subtle, the significant relationship with T stage and the improved fit of logistic regression models that include SII underscore its potential value as a complement to established prognostic markers. By integrating SII into a multifaceted assessment, clinicians may gain deeper insights into the host–tumor interplay, ultimately informing more nuanced patient management strategies and individualized treatment plans.

## 4. Discussion

This study adds to the growing body of evidence supporting SII as a prognostic marker in oncology. While SII’s role has been explored in various cancers, its application within a regional Romanian cohort of breast cancer patients provides a novel perspective. By integrating SII with clinicopathological factors, the findings underscore its potential to complement existing prognostic tools and enhance risk stratification in clinical practice [13].

A recent meta-analysis reinforced the prognostic value of inflammatory indices, including SII, across multiple cancer types [14]. Elevated SII levels were found to correlate significantly with advanced tumor (T) stage, suggesting a link between systemic inflammation and tumor burden. These findings align with the growing body of evidence that systemic inflammation plays a pivotal role in cancer progression, contributing to tumor growth, immune evasion, angiogenesis, and metastasis [1,8,15].

The significant association between SII and T stage highlights the importance of tumor size in driving systemic inflammatory responses. The observed correlations between tumor size and systemic inflammatory markers emphasize the dynamic interplay between tumor biology and the host immune response as larger tumors likely generate a higher inflammatory burden, which is reflected in elevated SII levels [16]. This relationship supports the hypothesis that SII serves as a surrogate marker for the tumor’s impact on the host’s immune-inflammatory milieu [17]. While no significant associations were observed between SII and other clinicopathological factors, such as N stage or hormonal receptor status, the strong relationship with tumor size suggests that SII captures specific aspects of tumor biology not necessarily linked to these parameters. This specificity may enhance its utility as an adjunctive prognostic tool, particularly in clinical scenarios where traditional markers are less informative.

Despite the significant associations observed between SII and advanced tumor stage, the lack of correlation between SII and certain clinicopathological factors, such as hormone receptor status, deserves further consideration. One possible explanation for these non-significant findings could be the inherent variability in tumor biology. Hormone receptor-positive breast cancers, particularly those with lower-grade or luminal A phenotypes, often exhibit less aggressive inflammatory profiles compared to triple-negative or HER2-enriched subtypes, which may dilute the overall relationship between SII and receptor status [7,9]. Additionally, the sample size in this study, while adequate for broader comparisons, may have been insufficient to detect subtle differences within smaller subgroups, such as HER2-positive or ki-67-high tumors [15,18]. Another plausible factor is the multifactorial nature of systemic inflammation, which may be driven by processes beyond tumor biology, such as co-morbidities, lifestyle factors, or the presence of subclinical infections [6]. These findings emphasize the need for further studies that explore the interaction between SII and breast cancer subtypes in larger, more diverse populations to uncover potential subgroup-specific patterns [19]. By addressing these nuances, future research can refine the understanding of SII’s prognostic value in breast cancer.

Interestingly, the observed weak inverse correlation between SII and age (r = −0.204, *p* = 0.043) provides additional context for the complex interplay between patient demographics and immune response. Younger patients demonstrated marginally higher SII levels, potentially reflecting more robust immune and inflammatory activity in younger individuals compared to older patients. This observation, while modest, warrants further exploration to elucidate whether age-specific variations in immune function influence systemic inflammation and cancer prognosis [5].

The receiver operating characteristic (ROC) curve analysis identified an optimal SII cutoff of 800, effectively stratifying patients into low- and high-risk groups. Patients with SII values above this threshold exhibited poorer prognostic features, such as advanced T stage, and were more likely to experience adverse outcomes. These results are consistent with previous studies demonstrating that elevated SII levels are associated with increased risks of recurrence, metastasis, and mortality [6,19]. By using a simple and accessible biomarker to stratify patients, clinicians may be better equipped to identify high-risk individuals who could benefit from closer monitoring, more aggressive treatment strategies, or participation in clinical trials.

The prognostic utility of SII was further validated through logistic regression models, which demonstrated that SII provides additional predictive value beyond standard clinicopathological markers. This reinforces the notion that SII captures unique aspects of systemic inflammation not fully accounted for by traditional variables. Importantly, SII is cost-effective and easily implementable in clinical settings, making it particularly valuable in resource-limited environments where advanced molecular diagnostics may be unavailable [9]

Systemic inflammation is increasingly recognized as a critical driver of cancer progression. Chronic inflammation can create a pro-tumorigenic microenvironment by promoting angiogenesis, increasing vascular permeability, and facilitating immune evasion [20]. This connection between inflammation and cancer was first proposed by Rudolf Virchow in the 19th century, who observed leukocytes in tumor tissues and hypothesized a link between inflammation and malignancy [21]. Modern research has confirmed and expanded on this hypothesis, illustrating how inflammatory mediators such as cytokines and chemokines contribute to tumor growth, metastasis, and immune suppression [21]. Furthermore, inflammatory cytokines released by tumors can stimulate the recruitment of neutrophils and platelets while suppressing lymphocyte activity, leading to elevated SII values. The observed correlation between SII and tumor size in this study aligns with the understanding that larger tumors exert greater systemic inflammatory effects, exacerbating the host’s immune dysregulation [9]

Despite the significant association of SII with advanced tumor stages, its lack of correlation with HER2-enriched and triple-negative subtypes warrants caution. These subtypes often exhibit aggressive behaviors but may not always trigger proportional systemic inflammatory responses, as evidenced by the current findings. This limitation may stem from sample heterogeneity or insufficient statistical power to detect subtle differences within subgroups. Stratifying larger cohorts by molecular subtype in future studies could uncover nuanced relationships, enhancing the clinical applicability of SII in personalized oncology. While SII captures certain inflammatory processes, it may not comprehensively reflect all aspects of the tumor’s interaction with the immune system. For example, the non-significant relationship between SII and ki-67, a marker of proliferation, suggests that SII may be more indicative of systemic effects than direct tumor cell activity [22]. The absence of BRCA mutation and neoadjuvant chemotherapy data limits the generalizability of these findings. Future studies incorporating these variables would provide more comprehensive insights into SII’s role across diverse patient subgroups. Further research is needed to elucidate the specific pathways linking SII to breast cancer outcomes [23].

This study’s limitations include its retrospective design, single-center setting, and limited sample size, which may affect generalizability. Additionally, unmeasured confounders such as lifestyle factors or subclinical inflammation could have influenced the findings. Prospective, multicenter studies are warranted.

Furthermore, the single-center setting may restrict the generalizability of the findings. Systemic inflammatory markers like SII can also be influenced by non-cancer-related factors, including comorbidities and lifestyle variables, which were not comprehensively accounted for in this analysis. To address these limitations, future studies should employ prospective, multicenter designs and incorporate broader patient populations to validate the findings. Additionally, exploring the impact of interventions targeting systemic inflammation, such as anti-inflammatory therapies, could provide insights into SII’s role in treatment response.

The generalizability of SII findings requires further validation in diverse populations, as systemic biomarkers are influenced by multi-level factors such as comorbidities and ethnicity [24]. Additionally, while the SII cutoff of 800 was derived through robust ROC analysis, its external validity requires confirmation in larger, prospective cohorts with diverse demographic and clinical characteristics.

Future studies should also investigate the biological mechanisms underlying the observed associations between elevated SII and poorer outcomes. For instance, it remains unclear whether interventions targeting systemic inflammation, such as anti-inflammatory agents or lifestyle modifications, could mitigate the adverse prognostic impact of high SII. Furthermore, integrating SII with other biomarkers, such as circulating tumor cells or genomic signatures, may provide a more comprehensive understanding of cancer prognosis and lead to the development of personalized therapeutic approaches [25].

The findings of this study have important implications for clinical practice. SII’s simplicity and cost-effectiveness make it a promising adjunct in resource-limited settings. Further studies should assess its role in guiding treatment decisions, including its integration into existing systems like the TNM classification. However, as a simple and cost-effective biomarker, SII has the potential to complement existing prognostic tools and inform treatment decisions in breast cancer. By identifying high-risk patients through SII stratification, clinicians can prioritize intensive surveillance and optimize resource allocation. Additionally, the accessibility of SII makes it particularly attractive for use in low-resource settings, where advanced diagnostic technologies may not be feasible. However, translating these findings into routine practice requires robust validation and standardization of SII cutoff values across different populations and healthcare systems. Future studies should integrate SII with other established biomarkers to enhance prognostic accuracy and provide a comprehensive assessment of patient risk [26].

## 5. Conclusions

This study demonstrates that the systemic immune–inflammation index (SII) shows potential as a prognostic marker in breast cancer, particularly for stratifying patients by risk and identifying associations with clinical outcomes [8]. However, the identification of an optimal SII cutoff (800) requires external validation, and its use as a predictive tool for guiding treatment decisions cannot be established based on the current data. Multivariate analysis highlighted its utility in understanding tumor-inflammation dynamics, but further research is needed to define its clinical applicability.

Importantly, SII offers several advantages. It is derived from routine blood tests, making it both cost-effective and widely applicable, particularly in resource-limited settings where advanced molecular diagnostics may not be feasible. By incorporating SII into existing prognostic frameworks, clinicians can improve the precision of patient counseling, prioritize high-risk individuals for intensive monitoring, and tailor therapeutic strategies to individual risk profiles.

Despite these promising findings, limitations such as the study’s retrospective design and single-center setting necessitate caution in generalizing the results. Prospective, multicenter studies are essential to validate the prognostic utility of SII across diverse populations and healthcare settings. While the study highlights SII’s association with prognostic outcomes, it does not establish predictive value. Further mechanistic and clinical investigations are necessary to validate its role in guiding treatment strategies. Furthermore, investigating the biological mechanisms linking systemic inflammation and cancer progression could enhance the understanding of SII’s role in breast cancer management [27].

Future research should also explore the integration of SII with other biomarkers, such as genomic signatures or tumor microenvironment profiles, to refine prognostic models further. As the landscape of breast cancer treatment evolves, SII has the potential to become a pivotal tool in advancing personalized oncology care.

## Figures and Tables

**Table 1:** Distribution of patient comorbidities, smoking history, and BMI according to age groups (<50 years and >50 years). Percentages represent the proportion of patients in each category. BMI classifications include normal weight, overweight, and obesity (Class I and II). This table highlights the prevalence of comorbid conditions and variations in BMI, reflecting demographic and lifestyle differences across the cohort.

Age Group	Diabetes (%)	Hypertension (%)	Smoking History (%)	BMI: Normal Weight (%)	BMI: Obesity Class I (%)	BMI: Obesity Class II (%)	BMI: Overweight (%)
<50 Years	57.1	57.1	61.9	28.6	23.8	14.3	33.3
>50 Years	49.6	48.9	49.6	31.4	29.9	15.3	23.4

**Table 2:** Age and SII values are presented as mean ± standard deviation. SII was calculated using the formula (Platelet Count × Neutrophil Count)/Lymphocyte Count. Additional parameters, including neutrophil, lymphocyte, and platelet counts, are also displayed, highlighting variability in inflammatory profiles across the cohort. The range of values indicates significant heterogeneity in systemic immune–inflammatory responses among patients.

	Age	SII	Neutrophils	Lymphocytes	Blood Palettes
Mean	62.797	708.028	4.684	1.966	268.386
Std. Deviation	10.850	448.591	1.583	0.634	68.632
Minimum	32.000	163.450	1.800	0.590	118.000
Maximum	85.000	3382.500	10.100	3.870	581.000

**Table 3:** Provenience indicates urban vs. rural residency of patients. T stage represents tumor size, and N affected nodes as per the TNM classification, HER2 (Human Epidermal Growth Factor Receptor 2), PR (progesterone receptor), and ER (estrogen receptors) statuses were classified as positive or negative based on immunohistochemistry results.

Variable	Mode	Interpretation
Provenience	2	Urban
T Stage	2	Tumor stage: T2
N Stage	0	No regional lymph node involvement
Histological Stage	2	Assumed discrete
HER2 (N/P)	0	Negative
PR Status	0	Negative
ER Status	0	Negative
Time-to-Event (Death) (<1, 1–2, >2)	3	>2 years

**Table 4:** Statistical significance for correlations was set at *p* < 0.05. SII demonstrated a significant positive correlation with T stage (r = 0.218, *p* = 0.01), indicating its association with tumor burden. No significant correlations were observed with HER2 (human epidermal growth factor receptor 2), PR (progesterone receptor), and ER (estrogen receptors) statuses.

Variable	Pearson’s r	*p*-Value
SII	-	-
T stage	0.218	0.01
N stage	0.004	0.96
Histological Stage	0.106	0.289
HER2(N/P)	0.031	0.826
ki-67	0.246	0.088
PR Status	−0.073	0.302
ER Status	−0.075	0.29

**Table 5:** Analysis of variance (ANOVA) was used to compare SII values across categorical variables. A significant effect was observed for T stage (F = 3.049, *p* = 0.019). Non-significant variables included N stage, histological grade, and ki-67 proliferation index.

Cases	Sum of Squares	df	Mean Square	F	*p*
T stage	2.473 × 10^6^	4	618,299.668	3.049	0.019
N stage	138,718.206	2	69,359.103	0.511	0.610
Histological Stage	233,695.339	2	116,847.670	0.861	0.444
ki-67 stage (<30%–>30%)	17,597.784	1	17,597.784	0.130	0.724
HER2(N/P)	44,437.270	1	44,437.270	0.328	0.576
Progesterone Receptor Status	39,110.021	1	39,110.021	0.288	0.600
Estrogen Receptor Status	43,122.580	1	43,122.580	0.318	0.582
Time-to-Event (Death)	26,598.802	1	26,598.802	0.196	0.665
Age Group (<50 and >50)	12,151.584	1	12,151.584	0.090	0.769

**Table 6:** The logistic regression model evaluates the likelihood of adverse outcomes (e.g., mortality) based on SII and clinicopathological variables. Model H_1_ (including SII) demonstrated improved fit compared to the null model (H_0_), with significant improvements in pseudo-R^2^ values.

Model Summary—Dead or Alive										
Model	Deviance	AIC	BIC	df	Χ^2^	*p*	McFadden R^2^	Nagelkerke R^2^	Tjur R^2^	Cox and Snell R^2^
H_0_	44.627	46.627	49.170	93						
H_1_	11.721	37.721	70.784	81	32.906	0.001	0.737	0.781	0.721	0.295

## Data Availability

The raw data supporting the conclusions of this article will be made available by the authors on request.

## Abbreviations

SII	Systemic Immune-Inflammation Index
ROC	Receiver Operating Characteristic
NLR	Neutrophil-to-Lymphocyte Ratio
PLR	Platelet-to-Lymphocyte Ratio
PR	Progesterone Receptor
ER	Estrogen Receptor
HER2	Human Epidermal Growth Factor Receptor 2
